# Haskap Berry Phenolic Subclasses Differentially Impact Cellular Stress Sensing in Primary and Immortalized Dermal Fibroblasts

**DOI:** 10.3390/cells10102643

**Published:** 2021-10-03

**Authors:** Lily R. Zehfus, Zoe E. Gillespie, Carla Almendáriz-Palacios, Nicholas H. Low, Christopher H. Eskiw

**Affiliations:** 1 Department of Food and Bioproduct Sciences, University of Saskatchewan, Saskatoon, SK S7N 5A8, Canada; lily.zehfus@usask.ca (L.R.Z.); c.almendariz@usask.ca (C.A.-P.); nicholas.low@usask.ca (N.H.L.); 2 Department of Biochemistry, Microbiology and Immunology, University of Saskatchewan, Saskatoon, SK S7N 5A8, Canada; zoe.gillespie@usask.ca

**Keywords:** phenolics, haskap berries, *Lonicera caerulea*, primary fibroblasts, intracellular free radicals, sirtuin 1

## Abstract

It is generally accepted that dietary phenolics from fruits are of significant importance to human health. Unfortunately, there is minimal published data on how differences in phenolic structure(s) impact biological pathways at cellular and molecular levels. We observed that haskap berry extracts isolated with ethanol:formic acid:water or phenolic subclass fractions separated using different concentrations of ethanol (40% and 100%) impacted cell growth in a positive manner. All fractions and extracts significantly increased population doubling times. All extracts and fractions reduced intracellular free radicals; however, there were differences in these effects, indicating different abilities to scavenge free radicals. The extracts and fractions also exhibited differing impacts on transcripts encoding the antioxidant enzymes (CAT, SOD1, GPX1, GSS and HMOX1) and the phosphorylation state of nuclear factor-κB (NF-κB). We further observed that extracts and fractions containing different phenolic structures had divergent impacts on the mammalian target of rapamycin (mTOR) and sirtuin 1 (SIRT1). siRNA-mediated knockdown of SIRT1 transcripts demonstrated that this enzyme is key to eliciting haskap phenolic(s) impact on cells. We postulate that phenolic synergism is of significant importance when evaluating their dietary impact.

## 1. Introduction

Diet has been reported to be the most influential environmental factor on human health and development with a recent research emphasis directed towards investigating the relationship(s) between the molecular structure(s) of chemical compounds in foods and their ability to impact different biological functions [1,2,3]. One field of particular interest is the study of how specific chemical compounds in the human diet target reactive oxygen species (ROS) [4,5]. ROS are highly reactive and harmful molecules produced through normal metabolic processes and environmental factors (e.g., electromagnetic radiation), and are directly involved in DNA damage, lipid peroxidation and the formation of advanced glycation end products, all of which contribute to cellular aging and the development of age-related disease(s) [6,7,8]. Our genome encodes proteins that act as defensive mechanisms to counteract ROS; however, these are not always sufficient, and dietary supplementation with compounds that scavenge ROS has been proposed to be beneficial. Significant research emphasis has been directed towards identifying chemical compounds within the diet that are able to both scavenge and provide protection against ROS formation [9]. Of particular interest are phenolics, a group of structurally diverse compounds comprised of at least one six carbon aromatic ring modified with at minimum one hydroxyl group and generally a covalently linked carbohydrate (e.g., d-glucose). Based on their structure, phenolics can readily scavenge free radicals and remain relatively stable, delaying the free radical propagation reaction. The majority of published research on dietary phenolics focus on fruit extracts, which are comprised of complex phenolic mixtures, and group them as either a single compound or class/subclass. However, given their structural diversity, it is logical that there would be significant differences in their impact on biological systems through the proteins/cellular components they interact with. It is also logical that the composition of these mixtures may work synergistically to promote increased radical scavenging as well as potential interaction with multiple pathways within cells simultaneously.

Haskap is an edible honeysuckle fruit that contains extremely high levels of phenolic compounds [10,11]. Recent in vivo and in vitro research on haskap extracts (i.e., complex phenolic mixtures) has indicated that their phenolics have properties ranging from anti-inflammatory to anticancer, and further claim that cyanidin-3-*O*-glucoside (C3G), as the major phenolic, is responsible for these effects [12,13]. We have developed a solid phase fractionation method using reverse phase chromatography coupled with ethanol:water as the mobile phase to successfully separate haskap berry phenolics based on their polarities. Phenolic class/subclass analysis was performed by high performance liquid chromatography with photodiode array detection (HPLC-PDA) with phenolic structural analysis within a fraction by high performance liquid chromatography-tandem mass spectrometry (HPLC-MS/MS).

The aim of this work was to determine if specific haskap phenolic extracts and fractions were able to influence cell growth and differentially impact important biological pathways beyond that of free radical scavenging. We treated primary fibroblasts (2DD) and telomerase immortalized human fibroblasts (NB1hT) with haskap phenolic extracts and fractions to evaluated their impact on cell growth, the ability to penetrate cells and to reduce levels of H_2_O_2_-induced free radicals. Although all extracts and fractions increased cell population doubling times, the fractions containing anthocyanins/hydroxycinammic acids/flavanols (40% ethanol) and flavanols/flavonols (100% ethanol) were most effective at reducing levels of anti-oxidant enzyme transcripts as well as reducing levels of the active form (phosphorylated) NF-κB. Transcripts encoding cytokines were consistently reduced by the 40% fraction, with the phenolic rich (PR) extract and C3G also resulting in significant reductions. C3G (23.7 µg/mL), the PR extract, and the 40 and 100% fractions all decreased levels of active phosphorylated mTOR. Although fractions containing complex phenolic mixtures were more effective at reducing anti-oxidant enzyme and cytokine transcripts as well as NF-κB and mTOR activity, we demonstrate that C3G and the PR extract had the greatest effect in activating the deacetylase, sirtuin 1 (SIRT1). These results indicate that although a single phenolic, such as C3G, has biological effects, the synergistic effect of haskap phenolics were found to have greater impact.

## 2. Materials and Methods

### 2.1. Haskap Berry Phenolic Extracts and Fractions

Haskap berries (Tundra variety, University of Saskatchewan Horticulture Field Laboratory,) were stored at −30 °C. A complete description of phenolic extract and fraction preparation can be found in [14,15]. The methods to produce extracts/fractions is briefly described—EFW extract: A fruit macerate was produced by mechanically blending the fruit at high speed for 2 min. To 25.00 ± 0.03 g of fruit macerate, 50.00 ± 0.03 g of ethanol:formic acid:water (EFW, 70:2:28% (*v*:*v*:*v*)) was added, the resulting mixture was covered and stirred at 700 rpm/4 ± 2 °C for 2 h. The mixture was then vacuum filtered, and the remaining sediment was removed and resuspended in 50.00 ± 0.03 g EFW. This mixture was stirred at 4 ± 2 °C for 2 h and vacuum filtered a second time. The filtrates were combined and quantitatively transferred to a 200 mL volumetric flask and brought to volume with EFW. Fractionation: 10 mL of dried and resuspended (in 5.0 mL of water) EFW extract was fractionated employing hydrated Amberlite^®^ XAD16N resin via column chromatography (50.0 cm × 2.0 cm) with sequential treatment with 60 mL of water (fraction 1), 60 mL of 20% (*volume*:*volume*; *v*:*v*) ethanol (fraction 2), 60 mL of 40% (*v*:*v*) ethanol (fraction 3), 60 mL of 70% (*v*:*v*) ethanol (fraction 4) and 60 mL of 100% ethanol (fraction 5). The 40 and 100% fractions were individually concentrated to dryness by rotary evaporation (35 °C). Phenolic rich (PR) extract: 10 mL of dried and resuspended (in 5.0 mL of water) EFW extract was fractionated employing hydrated Amberlite^®^ XAD16N resin via column chromatography (50.0 cm × 2.0 cm) with sequential treatment with 135 mL of water followed by 135 mL of 100% ethanol. The PR extract was concentrated to dryness by rotary evaporation (35 °C).

### 2.2. Cell Culture

A normal human fibroblast cell line (designated 2DD; [16]) and human telomerase immortalized fibroblasts (designated NB1hT) were grown in high glucose (4.5 mg/mL) Dulbecco’s Modified Eagle Medium (DMEM) supplemented with 10% (*v*:*v*) bovine calf serum (CS) and 1% (*v*:*v*) penicillin-streptomycin. Cells were plated at an initial density of 3000/cm^2^ and were not grown past 80% confluency. Cells were incubated at 37 °C in a humidified environment with 5% carbon dioxide (CO_2_). When cells reached ~70% confluency, they were passaged (i.e., harvested and reseeded) using TrypLE Express (Invitrogen, Carlsbad, CA, USA). Following counting (Tiefe 0.100 mm, 0.0025 mm^2^; Neubauer Improved, EM Sciences, Hatfield, PA, USA) cells were reseeded at 3000/cm^2^ using the total number of cells and surface area of the vessel. Cells from passage numbers 12 to 18 were used in experiments to ensure the use of ‘young’ primary cells. Culture media was changed every 2–3 days if passaging was not required. To prevent contamination, all handling of cell cultures (i.e., passaging, media changes, etc.) was performed in a sterile environment (Labconco, Kansas City, MO, USA).

### 2.3. Cell Treatments

Tundra variety phenolic extracts and fractions were individually freeze-dried and resuspended in dimethyl sulfoxide (DMSO) to produce stock solutions. Phenolic solutions were added to supplemented DMEM to achieve final concentrations of 5.0 and 50.0 μg phenolics/mL media (as determined by HPLC-PDA) for extracts and fractions and 2.4 and 23.7 μg/mL media for cyanidin-3-*O*-glucoside (C3G; Extrasynthese S.A. (Genay, France)) based on the amount of C3G theoretically present in the 5.0 and 50.0 μg/mL treatments, respectively. The volume of total DMSO added to create each treatment concentration was consistent and the vehicle control was prepared by adding that same volume of DMSO to supplemented DMEM. Note: DMSO was not used to increase cell membrane permeability but to solubilize treatment phenolics. To prevent the impact of DMSO on cell membrane permeability, volumes never exceeded 0.05% (*v*:*v*) in the media.

### 2.4. Cell Counts and Cell Viability

Cells (Section 2.2) were seeded in 6-well plates and left for 24 h to adhere to the plate surface. After this period, fresh DMEM was prepared, each containing the specific phenolic extract/fraction or C3G and added to cells. Cells were incubated at 37 °C for 72 h. Cells were collected (as per passaging) and counted. Population doubling times were calculated using the following formula: Population doubling time (h) = incubation time (h) × log(2) (final cell count/initial cell count). To assess cell viability, 50 μL of the above cell suspension was transferred to a 1.5 mL tube and mixed 1:1 with 0.4% (*v*:*v*) trypan blue dye and incubated for 7 min at room temperature (RT: 22.0 ± 2.0 °C). Stained and unstained cells were then counted using a hemocytometer, with dark blue stained cells considered nonviable. Percentage cell survival was calculated as follows: % cell survival = 100% × total counted cells−nonviable cells total counted cells.

### 2.5. MitoTracker Orange Labeling of Intracellular Free Radicals

Twenty-four hours after seeding cells onto coverslips (3000 cells/cm^2^), treatments (DMSO, 5.0 μg/mL and 50.0 μg/mL of the extracts/fractions and C3G at 2.3 and 24.7 μg/mL) were initiated as described (Section 2.4). Cells were then washed two times with serum-free DMEM. H_2_O_2_ (Sigma-Aldrich, St. Louis, MO, USA, Cat #: 216763) was added to serum-free DMEM media at a final concentration of 200 μM and incubated at 37 °C and 5% CO_2_ for 30 min. Cells were washed two times in serum-free media and then labelled with MitoTracker Orange CM-H2TMRos according to the manufacturer’s instructions (Molecular Probes, Cat #: M-7511). This is a reduced, non-fluorescent dye that stains mitochondria and becomes fluorescent when oxidized. After labelling, cells on coverslips were fixed with 3.7% formaldehyde in phosphate-buffered saline (PBS) for 10 min and permeabilized with 0.5% Triton X-100/PBS for 10 min at RT. Nuclei were counterstained with VECTASHIELD Mounting Medium with DAPI (Vector Laboratories, Cat #: H1200) and imaged by fluorescence microscopy. Images were collected at 60× magnification (Olympus X51 wide-field microscope) using identical imaging conditions and times. Signal intensity per unit area was measured using Image J.

### 2.6. Protein Extraction and Quantification

Cells were plated in 10 cm dishes and treated as described (Section 2.4). Following centrifugation (174 rcf for 5 min), the cell pellet was resuspended in 100 μL Laemmli lysis buffer (62.5 mM Tris-HCl pH 6.8, 2% sodium dodecyl sulfate (SDS; weight:volume; *w*:*v*), 10% glycerol (*v*:*v*), and 5% 2-mercaptoethanol (*v*:*v*)) containing protease inhibitor cocktail 2 and phosphatase inhibitor cocktail 2 in a ratio of 100:1:1 (*v*:*v*:*v*). Proteins were quantified employing UV spectroscopy at 280 nm using a NanoDrop™ 2000 Spectrophotometer (Thermo Fisher, Waltham, MA, USA).

### 2.7. Western Blotting

All cell protein extracts were diluted to the same concentration and denatured for 5 min at 95 °C prior to sample loading. Thirty to sixty μg per well were loaded on a 5% polyacrylamide stacking gel in conjunction with a 6–12% polyacrylamide resolving gel in 1× running buffer (25 mM Tris base, 192 mM glycine, and 0.1% SDS) at 125 V. Proteins were transferred from the gel to a nitrocellulose membrane (Bio-Rad Laboratories, Hercules, CA, USA) using 1× transfer buffer (25 mM Tris base, 192 mM glycine, and 20% methanol (*v*:*v*)) with a Trans-Blot^®^ SD semi-dry transfer cell (Bio-Rad Laboratories) at 25 V. Membranes were then blocked by incubating in 5.0% skim milk powder (SMP; *w*:*v*) in phosphate-buffered saline with TweenTM 20 (PBST; 8% sodium chloride (*w*:*v*), 0.2% potassium chloride (*w*:*v*), 0.76% disodium hydrogen phosphate (*w*:*v*), and 0.05% TweenTM 20 (*v*:*v*)) for 30 min at RT.

Membranes were incubated with primary antibody diluted in 5.0% SMP in PBST at 4 °C overnight: β-actin, 1:2000 (Abcam, Cambridge, MA, USA); phospho-NF-κB p65, 1:1000 (Cell Signaling Technology, Danvers, MA, USA); Nrf2, 1:200 (Santa Cruz Biotechnology, Dallas, TX, USA); phospho-mTOR, 1:500 (Santa Cruz Biotechnology); and SIRT1, 1:1000 (Abcam). Membranes were washed with ~15 mL of 5.0% SMP/PBST 3× followed by incubation with secondary antibody (goat anti-rabbit HRP and goat anti-mouse HRP; 1:2000 dilution) in 5 mL of 2.5% SMP/PBST at 4 °C overnight. Membranes were washed 3× with 5.0% SMP/PBST for 5 min, followed by PBST for 5 min, and PBS for 5 min. Protein bands were imaged using enhanced chemiluminescence (ECL) reagent (100 mM Tris-HCl pH 8.5, 0.2 mM *p*-coumaric acid, 1.25 mM luminol, and 0.1% hydrogen peroxide (H_2_O_2_ (*v*:*v*)). Following imaging, densitometry measurements were taken for each protein band using Image J software (64-bit Java 1.8.0_112). All protein measurements were normalized first to the loading control β-actin, and then to the control sample (untreated).

### 2.8. RNA Extraction

TRIzol™ reagent (1 mL) was added to pelleted treated cells and incubated at RT for 10 min, mixed with 200 μL chloroform and vortexed. Samples were incubated at RT for 5 min and then centrifuged at 21,130 rcf for 10 min at 4 °C. The aqueous phase was transferred to a new tube and the RNA precipitated by adding 1/10 volume of the aqueous phase of 3 M sodium acetate (pH 5.2) and an equal volume (to the aqueous phase) of isopropanol. Samples were incubated for 30 min on ice and then centrifuged at 21,130 rcf for 10 min at 4 °C. The supernatant was removed and the pellet was washed with 1 mL of cold 70% ethanol (*v*:*v*) and centrifuged (21,130 rcf for 10 min at 4 °C). Contaminating DNA was removed by DNase I treatment; RNA pellets were resuspended (88 μL nuclease free water, 1 μL RNaseOUT™, 10 μL 10× DNase I reaction buffer, and 1 μL DNase I) and incubated for 20 min at 37 °C. RNA was extracted from this mixture by adding 100 μL of acid phenol:chloroform (5:1 (*v*:*v*); pH 4.5) mixing and centrifugation (21,130 rcf for 10 min at 4 °C). RNA samples were dissolved in 30.5 μL of nuclease free water and 1 μL of RNaseOUT™ was added to inhibit RNase activity. Five μL of each sample was run on a 1% agarose gel and only those with clear 18 S and 28 S rRNA bands were used for cDNA synthesis. Sample RNA was quantified using a NanoDrop™ 2000 spectrophotometer at 260 nm and aliquots of 2 μg per 12.5 μL of nuclease free water were transferred to centrifuge tubes for cDNA synthesis. All sample aliquots were stored at −80 °C until cDNA synthesis. 

### 2.9. cDNA Synthesis and Reverse-Transcriptase-Quantitative PCR (RT-qPCR)

For cDNA synthesis, 1 μL of random primers (10 μM) and 1 μL deoxynucleotide triphosphate solution (10 mM) were added to RNA aliquots (thawed on ice) and incubated at 65 °C for 5 min. Samples were then placed on ice and 0.5 μL RNaseOUT™, 1 μL EasyScript RTase (200 U/μL), and 4 μL 5× RT buffer was added. Samples were then incubated at 25 °C for 10 min, 42 °C for 60 min, 85 °C for 15 min, and were then brought to 4 °C using a thermocycler. Samples were stored at −20 °C until analysis. RT-qPCR was performed in triplicate using 10 μL reactions prepared for each primer pair. Each reaction used 5 μL PerfeCTa^®^ SYBR^®^ Green SuperMix, 2 μL cDNA template, 1 μL nuclease free water, and 1 μL primer mix (forward and reverse each at 3 μM). Reactions were conducted using a Rotor-Gene^®^ Q qPCR (Qiagen, Germantown, MD, USA) with non-template controls. Melt curve analyses were performed to ensure that single products were produced in each reaction. Results were quantified using the ΔΔCt method to produce fold changes based on five normalizing genes: EFEMP2, FAU, FKBP10, PRDX5, and SPARC. Primers were designed using Primer 3 Software (version 0.4.0) and sequences (Supplementary material Table S1). 

Fold changes were calculated as follows:

ΔCt treatment sample = Ct target gene in treatment sample−Ct normalizing gene in treatment sample

ΔCt control sample = Ct target gene in control sample−Ct normalizing gene in control sample

ΔΔCt = ΔCt treatment sample−ΔCt control sample Fold change = 2−ΔΔCt

To eliminate outliers, quartile calculations were performed. Values that were 1.5 times higher than the upper band (1st quartile) or 1.5 times the value of the lower band (3rd quartile) were removed from the average and *p*-value calculations. 

### 2.10. SIRT1 Activity Assay

The 50.0 μg/mL concentration was used for all haskap phenolic treatments, and 23.7 μg/mL was used for C3G. Following treatment and harvesting, cells were resuspended in 100 μL non-denaturing lysis buffer (50 mM Hepes KOH (pH 7.5), 420 mM NaCl, 0.5 mM EDTA disodium salt, 0.1 mM egtazic acid, 10% glycerol (*v*:*v*)). Proteins were quantified employing UV spectroscopy at 280 nm using a NanoDrop™ 2000 spectrophotometer. Non-denaturing lysis buffer was used as a blank and a volume of 1.5 μL was used for each sample. Samples were then diluted with non-denaturing lysis buffer so as to be of the same concentration. All protein extracts were vortexed well and stored at −80 °C until analysis. SIRT1 activity was measured fluorometrically using an assay kit (Abcam, Cat #: ab156065). All reactions were prepared in a 96-well black microtiter plate. To test direct activation of SIRT1 by phenolics, an initial mixture was prepared of 20 μL water, 5 μL SIRT1 assay buffer, 5 μL 2 mM NAD, 5 μL 500 μg/mL each phenolic extract in DMSO (23.7 μg/mL for C3G), and 5 μL fluoro-substrate peptide. Developer was then added (5 μL) to each reaction and mixed well, followed by the addition of 5 μL recombinant SIRT1 (diluted 1 in 10 with water). The plate was immediately placed in the plate reader and fluorescence was measured at (350 nm excitation/460 nm emission) every 2 min for 60 min. Negative controls were run with no NAD and no SIRT1. 

To test SIRT1 activity in untreated lysates in the presence of all phenolic treatments, the initial mixture was prepared with 25 μL water (no NAD was added), 5 μL SIRT1 assay buffer, 5 μL 500 μg/mL phenolic extract in DMSO (23.7 μg/mL for C3G), and 5 μL fluoro-substrate peptide. After the addition of the developer, 5 μL of untreated cell lysate was added and fluorescence was measured. To test SIRT1 activity in haskap phenolic and C3G-treated lysates, the initial mixture was prepared with 25 μL water, 5 μL SIRT1 assay buffer, 5 μL Trichostatin A (10 μM in DMSO), and 5 μL 2 mM NAD. Developer was then added (5 μL) to each reaction and mixed well, followed by the addition of 5 μL treated cell lysates. Fluorescence was measured as described previously. 

Total reaction volumes in all cases were 50 μL. After fluorescence was measured for all wells, an arbitrary time point was selected and the fluorescence at that time was recorded for each reaction. These values were then normalized by dividing the fluorescence of the treated sample by the fluorescence of the untreated control sample and reported as normalized fluorescence (in arbitrary units).

### 2.11. siRNA Knockdown of SIRT1

Cells were grown in plates to ~80% confluence. Mixture A (50 μL of Lipofectamine^®^ 3000 reagent, 575 μL of Opti-Mem) was combined with mixture B (50 μL of siRNA at 10 μM (SIRT1 or control), 25 μL P3000TM reagent, and 550 μL Opti-Mem) and incubated at room temperature for 15 min. Cells were washed with 5 mL 1× PBS, followed by washing with 2 mL Opti-Mem per plate. The siRNA mixture in addition to another 1.5 mL of Opti-Mem. Following 4 h incubation at 37 °C, 2.5 mL DMEM (20% (*v*:*v*) bovine calf serum (CS) and 2% (*v*:*v*) penicillin-streptomycin) was added. Plates were incubated at 37 °C for 18 h and the media was aspirated and cells were treated as described (Section 2.4). Untreated cells exposed to control (scramble) siRNA and untreated cells exposed to SIRT1 siRNA were used as controls. The 50.0 μg/mL concentration was used for all haskap phenolic treatments, and 23.7 μg/mL was used for C3G. Following the 72 h treatment period, cells were removed by scraping from the plate surface and collected. Protein extraction and Western blotting were then performed as described previously. 

### 2.12. Chromatin Immuno-Precipitation

Following phenolic treatment, cells were fixed with 1% paraformaldehyde (Electron Microscopy Sciences, Cat #: 15714) for 10 min at RT, scraped in ice-cold PBS and pelleted. Pellets were resuspended in ChIP lysis buffer (1% SDS, 10 mM EDTA, 50 mM Tris-HCl pH 8.0) containing Protease Inhibitor Cocktail 2 (Sigma-Aldrich, Cat #: P8340) and Phosphatase Inhibitor Cocktail 2 (Sigma-Aldrich, Cat #: P5726). Following 10 min of incubation on ice, cells were sonicated into DNA fragments with 200–1000 bp in length. A total of 2.5 µg of mouse anti-Nrf2 antibody (Santa Cruz, Cat #: sc-365949) was added to sheared chromatin samples diluted 10 times in ChIP buffer (0.01% SDS, 1.1% Triton X100, 1.2 mM EDTA, 16.7 mM Tris-HCl pH 8.0 and 167 mM NaCl) containing Protease Inhibitor Cocktail 2 (Sigma-Aldrich, Cat #: P8340) and Phosphatase Inhibitor Cocktail 2 (Sigma-Aldrich, Cat #: P5726). A total of 2.5 µg donkey anti-mouse HRP (Jackson Scientific, Mansfield, UK, Cat #: 715-035-150) was used as non-specific antibody control. The mixture was incubated at 4 °C overnight with rotation, followed by binding to 50 µL Dynabead Protein A (Invitrogen, Cat #: 10006D) at 4 °C for 1 h. Samples were then treated with ChIP washing buffer I (0.1% SDS, 1% Triton X100, 2 mM EDTA, 20 mM Tris pH 8.0 and 150 mM NaCl), ChIP washing buffer II (0.1% SDS, 1% Triton X100, 2 mM EDTA, 20 mM Tris pH 8.0 and 500 mM NaCl) and ChIP washing buffer III (1 mM EDTA and 10 mM Tris-HCl pH 8.0). Samples were eluted with 500 µL of freshly made elution buffer (1% SDS and 0.1 M NaHCO_3_) for 1 h at RT. Crosslinks were reversed by adding 200 mM NaCl, 12.5 mM EDTA and 2 µL proteinase K (Invitrogen, Cat #: 25530049), followed by incubating at 65 °C for 5 h agitating at 900 rpm. DNA from each sample was extracted by phenol-chloroform and qPCR was performed following DNA extraction. Ten microliter reactions were set up using 5 μL PerfeCTa^®^ SYBR^®^ Green SuperMix for iQ (Quantabio, Beverly, MA, USA, Cat #: 95053-500), 1 μL ChIP DNA sample, 3 μL H_2_O and 1 μL of 3 μM forward and reverse ChIP primers. qPCR reactions were conducted using the Rotor-Gene Q qPCR machine (QIAGEN). All reactions for each gene were run in triplicate with non-template controls. ChIP-qPCR data was normalized by the percent input method. Standard error was calculated as a function of the standard deviations between triplicates.

## 3. Results

### 3.1. Haskap Berry Extracts and Fractions

To investigate the impact of haskap phenolics on biological pathways, we employed: (a) an EFW extract (5.0 and 50.0 μg/mL) containing all classes and subclass of phenolics in the haskap fruit, as this solvent system improves phenolic extraction over water alone (Supplementary material Figure S1A,B). In addition, this extract also contained ascorbic acid (antioxidant), carbohydrates, minerals (anti and pro-oxidant activities), organic acids, and soluble proteins; (b) a PR extract (>99% purity, 5.0 and 50.0 μg/mL) containing the all phenolic class and subclass composition of the fruit with non-phenolics (e.g., ascorbic acid) essentially removed; (c) a 40% aqueous ethanol fraction (5.0 and 50.0 μg/mL) containing mainly anthocyanins with hydroxycinnamic acids and flavanols as the next major subclasses; (d) a 100% ethanol fraction (5.0 and 50.0 μg/mL) containing flavanols and flavonols (essentially free of anthocyanins); and (e) cyanidin-3-*O*-glucoside (C3G) at concentrations of 2.4 and 23.7 μg/mL as this anthocyanin is the major phenolic present in haskap fruit. These concentrations matched those of this compound in the 5.0 and 50.0 μg/mL PR extract, respectively. 

### 3.2. Phenolic Extracts and Fractions Increased Population Doubling Times and Scavenged Intracellular Free Radicals

**Figure 1:**
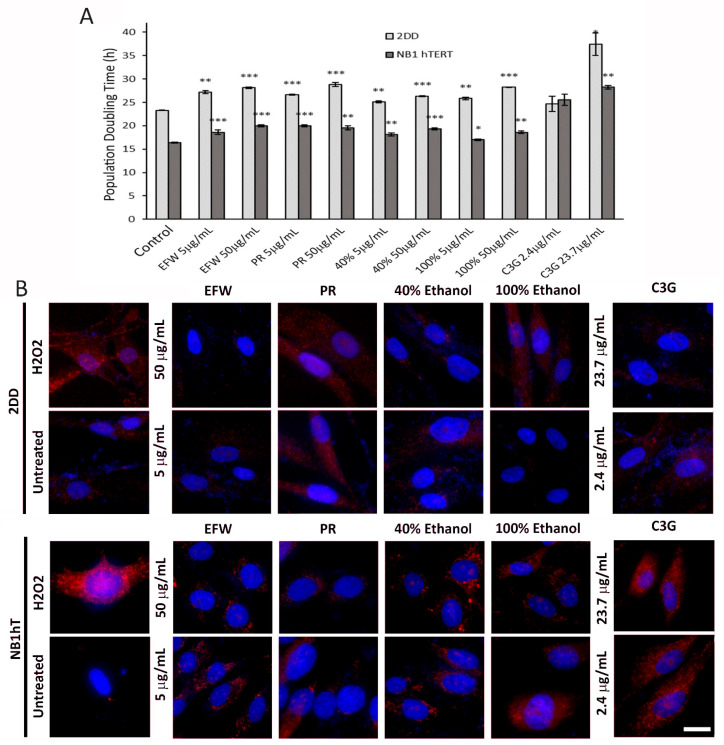
Haskap Phenolics Impact Fibroblast Growth and Reduce Free Radicals Intracellularly. (**A**) Population doubling times for 2DD (light grey bars) and NB1 hTERT (dark grey bars) fibroblasts after 72 h treatment with Tundra variety haskap phenolic extracts (5 μg/mL or 50 μg/mL) and C3G (2.4 μg/mL or 23.7 μg/mL). Treatment abbreviations: EFW, ethanol:formic acid:water extract; PR, phenolic rich extract; 40%, 40% ethanol fraction; 100%, 100% ethanol fraction; C3G, cyanidin-3-*O*-glucoside. *p*-values = * *p* < 0.10, ** *p* < 0.05, *** *p* < 0.01. (**B**) 2DD and NB1hT cells grown on coverslips were treated with 5 μg/mL or 50 μg/mL of EFW—ethanol:formic acid:water, PR—phenolic rich, 40%—40% ethanol fraction, 100%—100% ethanol fraction and C3G—cyanadin-3-*O*-glucoside (2.4 μg/mL or 23.7 μg/mL) for 72 h. Following this, cells were washed, exposed to 200 μM H_2_O_2_ for 30 min, fixed and stained with Mitotracker Orange (red) and DAPI (counterstain for DNA—blue). Images for each were collected using equivalent exposure times. Scale bar = 10 μm.

To evaluate the impact of haskap extracts and fractions on cells, we selected 2DD primary dermal fibroblasts and NB1hT telomerase immortalized dermal fibroblasts based on our previous assessment of the impact of the putative anti-aging compounds rapamycin [17] and metformin [18] as well as the oat specific polyphenolic compound avenanthramide C (AVNC) [19]. Our first aim was to determine if the phenolic extracts and fractions had observable impacts on cell growth in terms of population doubling times. With the exception of C3G at 2.4 μg/mL, all phenolic treatments significantly increased 2DD and NB1hT population doubling times, with mean increases of 4.3 and 4.1 h, respectively. C3G (23.7 μg/mL) exhibited the largest increase of 14.2 h for 2DD cells (Figure 1A; light and dark coloured bars) and 11.8 h for NB1hT cells, respectively. There was a greater impact found for the 100% fraction compared to the 40% fraction in 2DD cells; however, the 40% fraction had an almost identical impact compared to the 100% fraction in NB1hT cells, indicating that the phenolic composition of these fractions could account for differing effects in normal vs. immortalized cells. Population doubling time experiments at phenolic concentrations <5.0 and >50.0 μg/mL were also conducted; however, these did not exhibit impacts on cell doubling times beyond those observed at the concentrations studied (data not shown) and were not evaluated further. 

The observed change in population doubling times could result from increased cell death in response to treatment. Trypan blue assays were conducted and the percentage of viable cells within populations were determined with no observable changes in the number of viable cells (Figure S2). These assays showed that population doubling time increases were likely due to the slowing of cell growth. 

We have previously demonstrated via DPPH and ABTS assays that the aforementioned extracts and fractions scavenge free radicals in vitro [15]. Although previous reports found that haskap phenolic extracts reduced free radical production in cell culture systems [13], this may have been due to the interaction of phenolics with oxidative molecules (e.g., H_2_O_2_) outside of cells. To evaluate if haskap phenolics are interacting with intracellular radicals, 2DD and NB1hT cells were grown in the presence of the phenolic extracts and fractions for 72 h, washed with extract/fraction-free media to remove treatment phenolics that did not penetrate the cells, followed by treatment with H_2_O_2_ (200 μM, 30 min) to induce free radical formation. Cells were stained with MitoTracker^TM^ dye, imaged using fluorescence microscopy, with dye intensity within micrographs indicating the levels of intracellular free radicals in the treated vs. untreated controls (Figure 1B; representative images from multiple fields of cells are shown). Quantification of arbitrary intensity units of fluorescence signal demonstrate that in 2DD cells treated with the aforementioned extracts and fractions all demonstrated a decrease in dye intensity when compared to H_2_O_2_ treated cells. In NB1hT cells, all fractions and extracts reduced the level of intracellular free radicals as shown by decreased dye intensity; however, the C3G treatment was not as effective as the other treatments. Data is summarized in Supplemental Figure S3A,B for 2DD and NB1hT cells, respectively.

These results support the intracellular mechanistic role of phenolic structure(s) present in each of the extracts/fractions, either directly or indirectly, that resulted in decreased levels of oxidative stress. In addition, we observed that C3G alone had little to no impact on the levels of intracellular free radicals in NB1hT cells. This was contrary to that observed in normal 2DD cells and may indicate that specific phenolics/combinations could have differing impacts on primary non-immortalized vs. immortalized cells.

The authors recognize that the measurement of free radical scavenging in these experiments was not quantitative; however, these results do provide an assessment of the relationship between phenolic structure(s)/combinations in these extracts and fractions and intracellular free radical scavenging. We are confident that this scavenging occurred inside cells as excess phenolics in the media were removed that could have neutralized the H_2_O_2_ before entering cells. 

As the scavenging of free radicals results in decreased cellular oxidative stress [11,20,21], we tested the impact of haskap phenolic extracts and fractions on the production of intracellular radical scavenging enzymes. As such, RNA was extracted from 2DD and NB1hT cells treated with the haskap phenolic extracts, fractions and C3G. Following conversion of RNAs to cDNA libraries, quantitative reverse transcriptase-PCR (qRT-PCR) was performed for transcripts for catalase (CAT), superoxide dismutase 1 (SOD1), glutathione peroxidase 1 (GPX1), glutathione synthetase (GSS), and heme oxygenase-1 (HMOX1) (Table S3). All phenolic treatment values that resulted in less than a 3-fold change, either increased or decreased, were not considered relevant changes.

**Figure 2:**
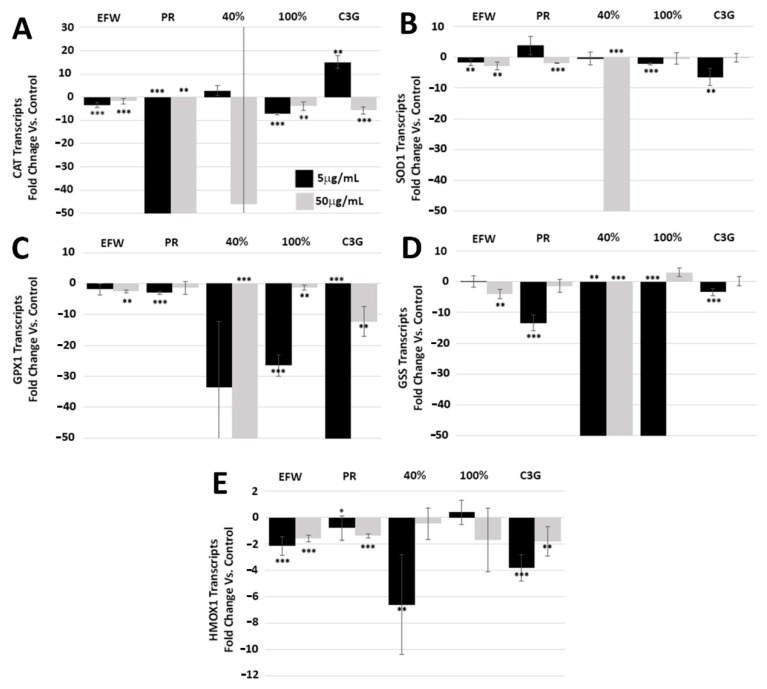
Specific Phenolic Fractions Decrease Antioxidant Transcript Levels. qRT-PCR quantification of transcripts encoding (**A**) CAT, (**B**) SOD1, (**C**) GPX1, (**D**) GSS and (**E**) HMOX1 following 72 h exposure of 2DD primary cells to EFW—ethanol:formic acid:water, PR—phenolic rich, 40%—40% ethanol fraction, 100%—100% ethanol fraction and cyanadin-3-*O*-glucoside at 5 μg/mL (black bars) and 50 μg/mL (grey bars). Graph values were cut off at 50-fold change vs. control. *p*-values = * *p* < 0.10, ** *p* < 0.05, *** *p* < 0.01 NOTE: quantified values are shown in Supplemental material Table S3.

CAT (Figure 2A) and SOD1 (Figure 2B) are cytosolic proteins involved with converting intracellular peroxides to water and molecular oxygen. The PR fraction had the greatest impact on reducing CAT transcripts at both concentrations (5.0 μg/mL −583.3-fold; 50.0 μg/mL −381.9-fold) but had no impact on SOD1. The greatest impact on SOD1 was seen with the 40% fraction (50.0 μg/mL −46.3-fold), with no impact on CAT. These results for the PR extract and the 40% fraction indicate that, although both CAT and SOD1 have similar roles, the downstream mechanisms regulating their transcript levels may not be the same and that the different subclasses of phenolics have different downstream effects. The 100% fraction reduced CAT transcripts at both concentrations (5.0 μg/mL −7.3-fold; 50.0 μg/mL −3.9-fold) but had no impact on SOD1. C3G decreased transcript levels of CAT at the higher concentration (23.7 μg/mL −5.7-fold) but resulted in an increase at the lower (2.4 μg/mL 15-fold). C3G further reduced SOD1 transcripts at the lower concentration (2.4 μg/mL −6.5-fold) but had no impact at the higher. The observed different changes to CAT and SOD1 transcripts by C3G when compared to the PR and the 40% fraction where this compound is the major phenolic; indicate possible synergistic phenolic roles/mechanisms in mediating regulation of these enzymes. 

GPX1 (Figure 2C) and GSS (Figure 2D) are both involved with the glutathione pathway [22,23] and were the most responsive to the 40% fraction, with GPX1 transcripts reduced at 50.0 μg/mL (−647.6-fold) and GSS reduced at both 5.0 μg/mL (−14,382.6-fold) and 50.0 μg/mL (−696.3-fold). The 100% fraction significantly reduced GPX1 transcripts (5.0 μg/mL −26.5-fold) and GSS (5.0 μg/mL −129.3-fold) as did C3G for GPX1 transcripts only at both concentrations (2.4 μg/mL −85.7-fold; 23.7 μg/mL −12.3-fold). These results indicate that phenolic structure, synergism and concentration impacted GPX1 and GSS, and are all important factors when evaluating their biological impact.

Although mainly associated with cleaving heme to form biliverdin [24], the HMOX1 enzyme plays an important role in modulating oxidative stress in non-erythrocyte cells and tissues. HMOX1 transcripts levels were not statistically responsive to most phenolic treatments; however, the 40% fraction (5.0 μg/mL) and C3G (2.4 μg/mL) induced −6.6-fold and −3.8-fold reductions in transcript levels, respectively (Figure 2E). The lack of responsiveness of HMOX1 to haskap phenolic treatments is interesting due to a previous report that has shown that the oat specific phenolic AVNC had a significant impact in up-regulating transcripts for this enzyme [19]. As AVNC is unique to oat, this supports our hypothesis that phenolic compounds have the potential to target specific biological pathways through their unique structures.

### 3.3. Phenolic Subclass Differentially Impacted NF-κB Activation

**Figure 3:**
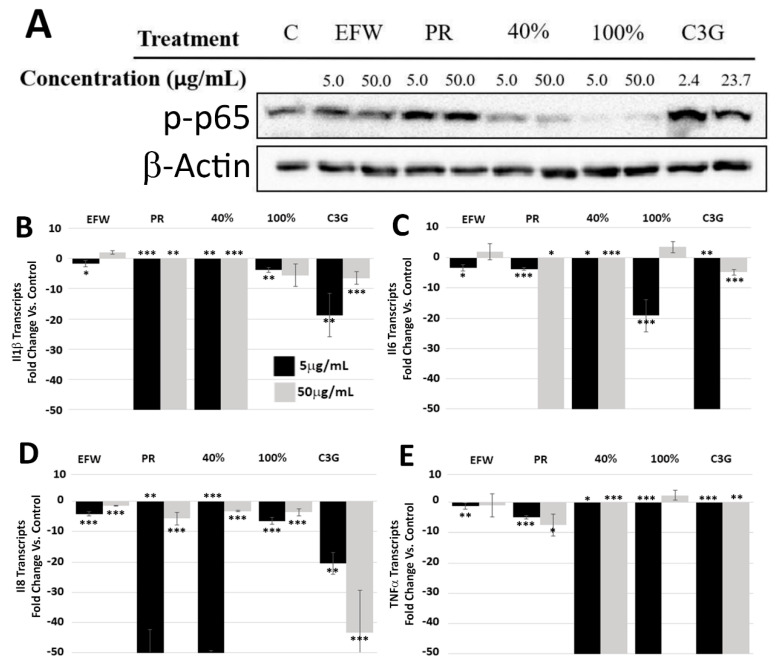
Specific Phenolics Fractions Reduce Levels of p65 Phosphorylation (p-p65) in Normal Dermal Fibroblasts and Decrease Cytokine Transcripts. (**A**) 2DD cells were treated for 72h with two concentrations (5 μg/mL or 50 μg/mL) of haskap phenolic extractions and fractions followed by Western blot analysis for p-p65 (top panel) and load control, β-actin (bottom panel). C—control, EFW—ethanol:formic acid:water, PR—phenolic rich, 40%—40% ethanol fraction, 100%—100% ethanol fraction. NOTE—this is one representative Western blot of two biological replicates quantified in Supplemental material Figure S3. qRT-PCR was used to probe cDNA libraries for Il1β (**B**), Il6 (**C**), Il8 (**D**) and TNFα (**E**) from 2DD cells exposed to 5 μg/mL (black bars) and 50 μg/mL (grey bars) of EFW, PR, 40% and 100% phenolic fractions as well as C3G (2.4 μg/mL and 23.75 μg/mL). Graph values were cut off after 50-fold changes. *p*-values = * *p* < 0.10, ** *p* < 0.05, *** *p* < 0.01 NOTE: quantified values are shown in Supplemental material Table S4.

Nuclear factor-κB (NF-κB), a heterodimer of the p50 and RELA/p65 proteins, is a key transcriptional regulator mediating oxidative stress. It has been previously demonstrated that phenolics can reduce NF-κB activity by decreasing the phosphorylation of the p65 subunit (p-p65) [4,20,25]. To determine if haskap phenolic treatments had differential effects on p-p65 levels, we performed Western blotting of whole cell protein extracts from 2DD cells treated for 72 h (Figure 3A). The 40% (anthocyanins/hydroxycinnamic acids/flavanols subclasses) and 100% (flavanols/flavonols subclasses) fractions had a greater impact on reducing p-p65 levels when compared to all other treatments, including C3G, indicating synergistic phenolic structure effects within these fractions. We further evaluated if this decrease in p-p65 was concomitant with transcript levels regulated by NF-κB by employing cDNA libraries generated from cells treated with 40 and 100% fractions. qRT-PCR was used to determine the transcript levels of four genes regulated by NF-κB; interleukin (Il) 1β, Il6, Il8 and tumor necrosis factor α (TNF α (Figure 3B). Fold change values were generated by taking the raw Ct values from the aforementioned transcripts and individually comparing them to Ct values from transcripts from five selected control genes (*SPARC, FAU, FKBP10, PRDX5* and *EFEMP2*) based on previously published RNAseq data [26] (quantified values presented in Supplementary material Table S4A–D). The EFW extract exhibited relatively small decreases in transcript levels compared to the PR extract and fractions, and only at the lower concentration. The PR extract resulted in significant decreases in all transcripts at both concentrations, indicating that this extract was more effective than EFW which contained non-phenolic molecules, such as carbohydrates. The 40% fraction exhibited the greatest reduction in transcripts at both concentrations. The 100% fraction reduced all transcript levels but only at the lower concentration (5.0 μg/mL); however, it was not as effective as the 40% fraction. In addition, the 100% fraction at 50.0 μg/mL only reduced Il8 transcripts. This demonstrates that the differences in phenolic structures present in the 40 vs. 100% fractions had different impacts on decreasing cytokines. The C3G treatment produced decreases in all cytokine transcript levels at both concentrations. As this compound is present in both the PR and 40% fraction but not the 100% fraction, it may account for the decreased effectiveness of the latter. However, as the 40% fraction out-performed C3G in these experiments, the impact of phenolic synergism is further supported. Finally, the 100% fraction was most effective at lowering the levels of phosphorylated/active NF-κB but least effective at reducing cytokine transcripts. This observation suggests that mechanisms other than NF-κB impact cytokine transcripts and are less responsive to the phenolics present in the 100% fraction compared to the other treatments. 

### 3.4. Phenolic Subclass Impacts Cellular Pathways That Promote Cellular Health

**Figure 4:**
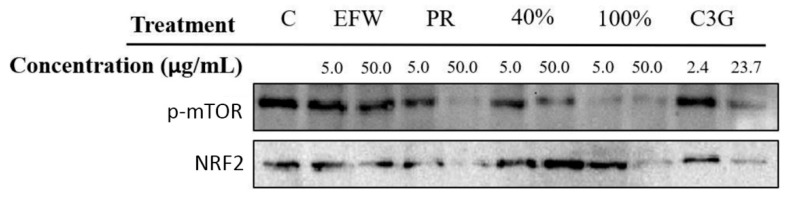
Specific Phenolic Fractions Reduce mTOR Phosphorylation and Increase NRF2 Protein Levels. Western blot analysis for phosphorylated mTOR (phospho-mTOR) (top panel) and NRF2 (bottom panel) protein following 72h exposure of 2DD cells to phenolic fractions at 2 concentrations (5 μg/mL or 50 μg/mL). C—control, EFW—ethanol:formic acid:water, PR—phenolic rich, 40%—40% ethanol fraction, 100%—100% ethanol fraction. Equivalent amounts of protein were loaded into each blot and probed for phospho-mTOR (residue 2448) and NRF2.

The mammalian target of rapamycin complex I (mTORC1) is associated with cellular health and lifespan, and numerous naturally occurring compounds impact mTORC1 activity [1,27,28]. The mTOR protein is the catalytic kinase subunit of mTORC1 and is activated by phosphorylation (p-mTOR). When conditions become unfavorable, such as depletion of cellular energy or nutrients, p-mTOR is dephosphorylated (mTOR) leading to changes in downstream pathways involved with cellular maintenance and repair, such as autophagy [29]. To determine if phenolic treatments impacted p-mTOR levels, Western blotting of 2DD whole cell protein extracts were conducted (Figure 4: top panel). Experimental results for p-mTOR levels were: (a) no change for the EFW fraction at both concentrations; (b) a large decrease for the PR fraction at 50.0 μg/mL and a small decrease at 5.0 μg/mL; (c) for the 40% fraction, a similar pattern was observed to that of the PR extract; (d) a large decrease was observed for the 100% fraction at both concentrations; and (e) C3G showed a reduction at 23.7 μg/mL only. The observed differences on p-mTOR activation as shown by the 40 and 100% fractions further support our hypothesis that phenolic structure(s) are responsible for eliciting contrasting biological responses.

The nuclear erythroid 2-like-factor 2 (NRF2) protein has been shown to be activated by dietary compounds such as sulforophane [28] and is a key regulator of several genes involved with cellular health, including *HMOX1*. Although these phenolic treatments did not elicit changes in HMOX1 transcript levels (Figure 3E), this result alone does not eliminate the possibility of a change in NRF2 protein levels/activity. To evaluate the impact of the phenolic treatments on NRF2 protein levels, we performed Western blotting of 2DD whole cell protein extracts following 72 h of treatment (Figure 4, bottom panel; quantified values in Supplementary material Figure S5A). We observed that the 40 (5.0 μg/mL and 50.0 μg/mL) and 100% (5.0 μg/mL) fractions increased NRF2 protein levels when compared to untreated controls. All other phenolic treatments had no significant effect on NRF2 protein levels. 

The lack of HMOX1 induction appears contradictory to that of the Western blot data for NRF2 in the 40 and 100 % fractions. However, NRF2 is sequestered in the cytoplasm under non-stress conditions by KEAP1 and this indicates that although there may be more protein in response to phenolic treatments, it did not access gene promoters in these assays. To test if the observed increase in NRF2 protein levels in 2DD cells induced by the 40 and 100% fractions lead to increased promoter binding, we performed chromatin immuno-precipitation (ChIP) assays followed by qPCR quantification for five genes known to have NRF2 binding sites: FOS, IL-8, Il-6, NQO-1 and IL-1β [19,30]. No increase in NRF2 promoter occupancy was observed (Supplementary material Figure S5B), which may be explained by NRF2 not being released from KEAP1, or that it is bound to other regions of the genome and not with these specific promoters. 

### 3.5. Sirtuin 1 Is Indirectly Impacted by Haskap Phenolics But Is Required for Downstream Effects

**Figure 5:**
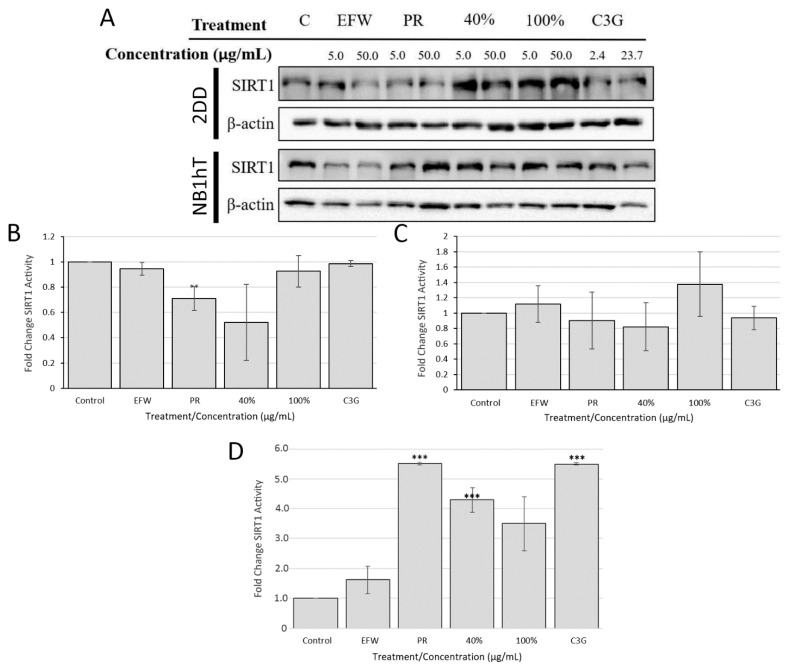
SIRT1 Protein and Enzymatic Activity Increase in Response to Haskap Phenolic Fractions. (**A**) 2DD (top two panels) and NB1hT (bottom two panels) cells were exposed to 2 concentrations (5 μg/mL or 50 μg/mL) of haskap phenolic fractions (C—control, EFW—ethanol:formic acid:water, PR—phenolic rich, 40%—40% ethanol fraction, 100%—100% ethanol fraction) for 72 h and total cellular proteins harvested. Western blots of equal protein for each samples were probed for SIRT1; β-actin westerns were used for load controls. SIRT1 activity assays were performed using DMSO mock controls, EFW, PR, 40%, 100% and C3G with either recombinant SIRT1 (**B**), treatment of cell lysates following isolation (**C**) or performed on cell lysates that were treated for 72 h prior to isolation (**D**). Activity is measured in arbitrary intensity units of fluorescence. Error bars are the S.E.M. *p*-values = ** *p* < 0.05, *** *p* < 0.01.

Sirtuin1 is a deacetylase enzyme targeting several proteins/pathways, including those involved with mediating cellular stress and promoting cellular and organismal lifespan such as NF-κB, mTOR and NRF2 [31]. Several phenolics [32,33,34,35], including resveratrol [36,37,38], have been reported to influence SIRT1 protein levels. To determine if these phenolic treatments had an impact on SIRT1 protein levels, we performed Western blotting for SIRT1 on whole cell protein extracts isolated from two independent biological replicates of 2DD and NB1hT cells treated for 72 h (Figure 5A). The modest increases/decreases in protein levels observed in this study (data not shown), which were below our statistical significance threshold (</> 3-fold), were much lower than those reported previously for haskap extracts [33]. Possible reasons for these differences include: (a) how the specific extracts were generated and quantified in the aforementioned study; (b) the use of different cell types/lines, one of which was primary and as such would result in variance; (c) treatment times; and (d) the haskap varieties used and their maturity, and the agronomic and environmental growth conditions used, both of which impact fruit phenolic profiles and concentrations.

The levels of SIRT1 are important to cellular function; however, as with our results for NRF2 responsiveness to haskap phenolic treatments, this may not equate to changes in enzymatic activity. In order to test the impact of phenolic treatment on SIRT1 activity, we initially examined if they directly interacted with recombinant SIRT1 in cell free assays. For all SIRT1 activity assays, the 50.0 μg/mL concentration for extracts/fractions and 23.7 μg/mL for C3G were evaluated. No significant increases in recombinant SIRT1 activity (i.e., no repression of SIRT1) with these treatments were observed (Figure 5B). We further evaluated if these phenolic treatments could interact directly with cellular components by their mixing with total 2DD cell lysates (Figure 5C). The SIRT1 activity assays did not result in a significant change, indicating that endogenous cellular SIRT1 did not directly interact with these phenolics. Finally, the impact of these phenolic treatments on SIRT1 activity for 2DD cells was examined following 72 h treatment (Figure 5D). Significant increases in SIRT1 activity were observed in response to: the PR extract (5.5-fold); the 40% (4.3-fold) fraction; and C3G (5.5-fold). The equivalent impacts on SIRT1 activity for the PR extract and C3G indicates that this phenolic [anthocyanin] is most likely responsible for the observed indirect activation of SIRT1 function. This conclusion is based on the fact that the concentration of this phenolic in the PR extract was equivalent to the C3G concentration used (i.e., 23.7 μg/mL). Although C3G is also present in the 40% fraction, it is at a lower concentration than in the PR extract, which explains the lower increase in SIRT1 levels for this phenolic treatment. Although, SIRT1 levels have been evaluated previously, to the best of our knowledge this is the first report of SIRT1 activity in response to haskap fruit extracts and fractions, and that this is an indirect interaction requiring intact cells.

**Figure 6:**
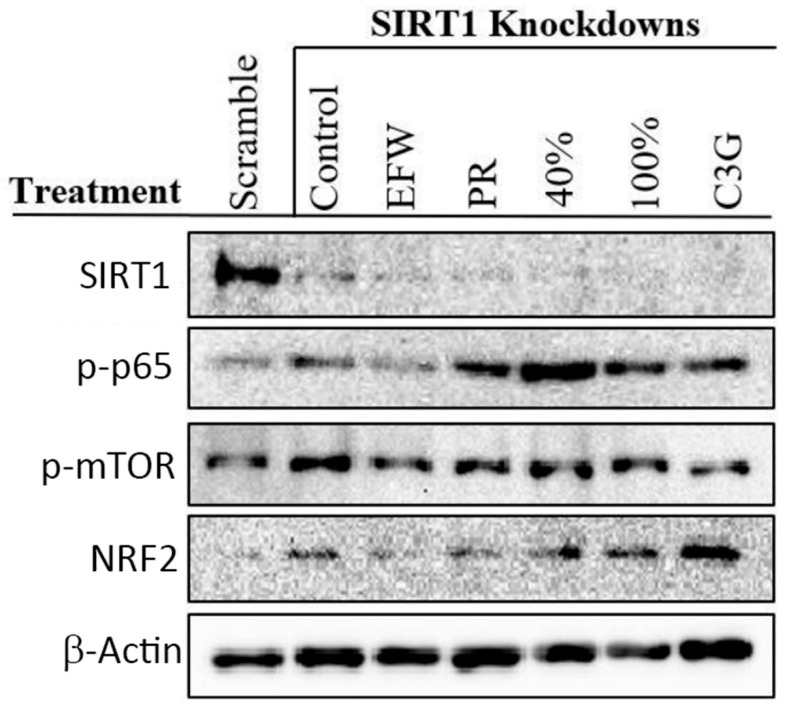
Knockdown of SIRT1 Inhibits Haskap Phenolic-Mediated Changes p-p65 and p-mTOR Levels but not NRF2 Protein Levels. 2DD cells were exposed to phenolic fractions for 72 h following siRNA-mediated knockdown of SIRT1. Equivalent whole cell protein lysates were blotted for SIRT1 (top row), p-p65 (2nd row), p-mTOR (3rd row) and NRF2 (4th row). β-actin was used as a load control and served a baseline for quantification.

As SIRT1 has several downstream targets, such as p-p65 and p-mTOR, we performed assays to determine if SIRT1 activity was required for specific phenolic extracts and fractions to impact these targets. We transiently transfected short interfering RNA (siRNA) for SIRT1 into 2DD cells, which resulted in the degradation of messenger RNAs encoding SIRT1 with concomitant knocking-down of SIRT1 levels. Phenolic treatment(s) of transfected 2DD cells were unable to rescue/restore SIRT1 levels (Figure 6, 1st row). We demonstrated (Figure 3) that phenolic treatments reduce levels of p-p65; however, in cells where SIRT1 had been knocked down, PR, 40 and 100% fractions (all treatments at 50.0 μg/mL) and C3G (23.7 μg/mL) increased levels of p65 phosphorylation (Figure 6, 2nd row). Furthermore, p-mTOR levels remained constant in SIRT1 knockdown cells (Figure 6, 3rd row) while inducing slight increases in NRF2 levels with all phenolic treatments (Figure 6, 4th row). This indicates that NRF2 protein levels and as-of-yet unidentified functions occur independently of SIRT1. We also noted that SIRT1 knockdown in 2DD cells with phenolic treatment failed to induce significant changes in population doubling times, with the exception of a slight but significant (2 h) increase in response to the 100% fraction (Supplementary materials Figure S6). These results indicate that SIRT1 plays a central role in mediating the response of 2DD cell growth in response to haskap phenolic treatment. 

## 4. Discussion

The aim of the research was to investigate potential differential impacts of haskap berry (Tundra variety) phenolic subclasses (i.e., structure) on cell growth and biological pathways linked to oxidative stress. To accomplish this, primary and immortalized dermal fibroblasts were treated with haskap phenolic extracts (EFW and PR) and fractions (40 and 100%), and cyanidin-3-*O*-glucoside (C3G) at two concentrations. We found that all phenolic treatments increased cell population doubling times resulting from a slowing of cell growth and not a change in cell viability. Intracellular (NB1hT cells) free radical scavenging by all phenolic treatments was observed by fluorescence microscopy imaging via MitoTracker^TM^ dye, although there was variation in how these extracts/fractions performed. The relationship between phenolic treatment and five intracellular radical scavenging enzymes (CAT, SOD1, GPX1, GSS, HMOX1) was followed by quantitative reverse transcriptase-PCR (qRT-PCR) on transcripts from cDNA libraries. With the exception of HMOX1, all phenolic treatments elicited decreases in transcripts for these radical scavenging enzymes, with the 40% fraction being the most potent. In addition, we found that this fraction had the greatest impact on the NF-κB and mTOR stress sensing pathways, resulting in decreased levels of pro-inflammatory cytokines. Results from SIRT1 and SIRT1 knock down activity experiments illustrated the importance of C3G in mediating SIRT1 responsiveness, as the 100% fraction did not elicit a significant response. However, these assays demonstrate that the interaction between C3G and SIRT1 is likely indirect as C3G and the fractions had no impact on recombinant SIRT1 in cell-free environments. This is one of the limitations of the current study, we cannot be exact with which specific phenolics, or combinations of phenolics are having the effect and which specific proteins/pathways they are directly interacting with. Future studies will aim at identifying which pathways are directly impacted as well the specific phenolic structures that mediate these responses.

Based on experimental results, we postulate that: (a) phenolic synergy (i.e., structure) plays an important role in mediating cellular stress as the PR extract (containing all haskap phenolics), 40% fraction (anthocyanins/hydroxycinnamic acids/flavanols), and 100% fraction (flavanols/flavonols) outperformed C3G in assays measuring intracellular free radicals and antioxidant transcripts; (b) an optimum phenolic concentration is required; (c) multiple parallel biological pathways may be activated or repressed in response to specific phenolic treatment.

## Supplementary Materials

The following are available online at https://www.mdpi.com/article/10.3390/cells10102643/s1: Figure S1: Separation strategy to Generate Haskap Phenolic Fractions and their Composition, Figure S2: Phenolic Fractions and Extracts do not Result in Numbers of Trypan Blue Cells, Figure S3: Phenolic Extracts and Fractions Decrease Intracellular Free Radicals, Figure S4: 2DD Cell p-p65 Normalization to Control Quantification Following Exposure to Haskap Phenolic Fractions, Figure S5: NRF2 Protein Levels Demonstrate Responsiveness to Haskap Phenolics but did not Increase DNA binding, Figure S6: NF-κB/p-p65 is No Longer Responsive to Phenolics Following SIRT1 Knockdown in Immortalized Fibroblasts, Table S1: qPCR Primers used for Gene Expression Analysis, Table S2: Phenolic Compounds Identified in Haskap Berry Fractions, Table S3: Haskap Berry Extract and Fraction Impacts on Antioxidant Enzyme Transcripts, Table S4: Haskap Berry Extract and Fraction Impacts on Cytokine Transcripts.
