# Supplementary material for: Haskap Berry Phenolic Subclasses Differentially Impact Cellular Stress Sensing in Primary and Immortalized Dermal Fibroblasts
Source: Cells. 2021 Oct 3;10(10):2643. doi: 10.3390/cells10102643 (PMC8534008; doi:10.3390/cells10102643)
Supplement: Supplementary file 1 [file cells-10-02643-s001.zip › cells-1353005-SI.pdf]

Supplementary Table S1. qPCR Primers used for Gene Expression Analysis.

RT-qPCR primer sequences	Forward Primer Sequence (5'→3')	Reverse Primer Sequence (5'→3')
EFEMP2	CGGTTCTCAGAGACCTGGATG	GCCCAAACCTGTGTCAACTTC
FAU	CGCATGCTTGGAGGTAAAGTC	TTCTCCTGTTTGGCCACCTTA
FKBP10	GCCGTGCTAATCTTCAACGTC	GGTGGTCTCATTGCAGGTCTC
PRDX5	GGGGTGGAGGAAGTAATCTG	GCATAGTGAAGGCCCTGAATG
SPARC	TACATCGGGCCTTGCAAATAC	GGTGACCAGGACGTTCTTGAG
CAT	TGCAAGCTAGTGGCTTCAAAA	TCCAATCATCCGTCAAAACAA
GPx1	CCTCCCCTTACAGTGCTTGTT	GAGAAGGCATACACCGACTGG
GSS	AGCTTTCCATCTGAGGACCAG	TCCTATCCCAAGTCAGGCACT
HMOX1	AAAGGAGGAAGGAGCCTATGG	AGCTGCCACATTAGGGTGTCT
SOD1	GGCAAAGGTGGAAATGAAGAA	GGGCCTCAGACTACATCCAAG
IL-1β	GCTACGAATCTCCGACCACCA	AACCAGCATCTTCCTCAGCTTG
IL-6	CGTCCGTAGTTTCCTTCTAGCTT	CAAAGGAGGACCTTGTGGCA
IL-8	TGCAGTTTTGCCAAGGAGTG	TGATAAATTTGGGGTGGAAAGG
TNF-α	CAATGGCGTGGAGCTGAGAG	TCTGGTAGGAGACGGCGATG

Supplementary Table S2. Phenolic Compounds Identified in Haskap Berry Fractions.

ferulic acid*	quercetin-vicianoside	quercetin-3-*O*-rutinoside*	quercetin-3-*O*-glucoside*
quercetin-3-*O*-galactoside*	quercetin-pentoside	kaempferol-rutinoside	isorhamnetin-3-*O*-rutinoside*
kaempferol-hexoside	isorhamnetin-3-*O*-glucoside*	quercetin-acetyl-hexoside	phloridzin*
quercetin-hexoside	isorhamnetin-acetyl-hexoside		

Supplementary Table S3. Haskap Berry Extract and Fraction Impacts on Antioxidant Enzyme Transcripts.

CAT	Average	S.E.M.	p-Value
EFW 5μg/mL	-3.4	0.4	4.37E-05
EFW 50μg/mL	-1.7	0.2	2.91E-05
PR 5μg/mL	-548.3	41.1	1.09E-05
PR 50μg/mL	-381.9	160.6	5.45E-02
40% 5μg/mL	2.7	2.3	6.10E-01
40% 50μg/mL	-46.3	26.8	1.00E-01
100% 5μg/mL	-7.3	1.3	2.75E-04
100% 50μg/mL	-3.9	1.7	2.61E-02
C3G 2.4μg/mL	15.0	5.5	1.92E-02
C3G 23.7μg/mL	-5.7	1.8	2.77E-03

SOD1	Average	S.E.M.	p-Value
EFW 5μg/mL	-1.7	1.0	3.21E-02
EFW 50μg/mL	-2.8	1.3	2.69E-02
PR 5μg/mL	3.8	2.9	3.67E-01
PR 50μg/mL	-1.8	0.1	4.07E-07
40% 5μg/mL	-0.5	2.1	5.14E-01
40% 50μg/mL	-2320.1	153.7	1.47E-06
100% 5μg/mL	-2.1	0.4	2.30E-04
100% 50μg/mL	-0.4	1.8	4.47E-01
C3G 2.4μg/mL	-6.5	2.8	1.76E-02
C3G 23.7μg/mL	-0.2	1.5	4.02E-01

GPX1	Average	S.E.M.	p-Value
EFW 5μg/mL	-1.9	1.8	1.58E-01
EFW 50μg/mL	-2.5	0.4	3.60E-05
PR 5μg/mL	-3.0	0.4	1.56E-04
PR 50μg/mL	-1.3	2.1	3.12E-01
40% 5μg/mL	-33.5	21.2	1.54E-01
40% 50μg/mL	-674.6	37.7	2.73E-06
100% 5μg/mL	-26.5	3.4	1.81E-04
100% 50μg/mL	-1.3	0.9	3.75E-02
C3G 2.4μg/mL	-85.7	22.3	4.61E-03
C3G 23.7μg/mL	-12.3	4.8	2.48E-02

GSS	Average	S.E.M.	p-Value
EFW 5μg/mL	0.0	1.9	6.10E-01
EFW 50μg/mL	-4.0	1.6	1.16E-02
PR 5μg/mL	-13.5	2.6	1.38E-03
PR 50μg/mL	-1.3	2.1	3.12E-01
40% 5μg/mL	-14382.6	4890.4	2.59E-02
40% 50μg/mL	-696.3	46.3	1.45E-06
100% 5μg/mL	-129.3	14.8	1.17E-04
100% 50μg/mL	3.0	1.4	2.58E-01
C3G 2.4μg/mL	-3.4	1.1	2.00E-03
C3G 23.7μg/mL	0.1	1.5	5.66E-01

HMOX	Average	S.E.M.	p-Value
EFW 5μg/mL	-2.2	0.7	4.46E-03
EFW 50μg/mL	-1.6	0.2	2.84E-05
PR 5μg/mL	-0.8	0.9	8.10E-02
PR 50μg/mL	-1.4	0.1	2.60E-06
40% 5μg/mL	-6.6	3.8	8.00E-02
40% 50μg/mL	-0.5	1.2	2.39E-01
100% 5μg/mL	0.4	0.9	5.30E-01
100% 50μg/mL	-1.7	2.4	2.90E-01
C3G 2.4μg/mL	-3.8	1.0	1.00E-02
C3G 23.7μg/mL	-1.8	1.1	2.00E-02

Supplementary Table S4. Haskap Berry Extract and Fraction Impacts on Cytokine Transcripts.

IL1β	Average	S.E.M.	p-Value
EFW 5μg/mL	-1.7	1.1	5.19E-02
EFW 50μg/mL	2.0	0.6	2.79E-01
PR 5μg/mL	-126.1	25.7	1.29E-03
PR 50μg/mL	-138.3	58.3	2.71E-02
40% 5μg/mL	-144.6	48.0	2.31E-02
40% 50μg/mL	-1169.5	86.5	2.81E-06
100% 5μg/mL	-3.7	0.8	1.67E-02
100% 50μg/mL	-5.6	3.7	1.50E-01
C3G 2.4μg/mL	-18.7	7.1	1.59E-02
C3G 23.7μg/mL	-6.5	2.1	3.70E-03

Il6	Average	S.E.M.	p-Value
EFW 5μg/mL	-3.3	1.0	8.76E-02
EFW 50μg/mL	2.0	2.6	7.02E-01
PR 5μg/mL	-3.7	0.4	3.04E-05
PR 50μg/mL	-26513.7	21940.3	5.52E-02
40% 5μg/mL	-1298.0	991.1	8.68E-02
40% 50μg/mL	-143.6	9.4	6.35E-06
100% 5μg/mL	-19.1	5.4	9.38E-03
100% 50μg/mL	3.6	1.8	1.01E-01
C3G 2.4μg/mL	-167.5	45.2	1.02E-02
C3G 23.7μg/mL	-4.8	0.9	1.79E-03

Il8	Average	S.E.M.	p-Value
EFW 5μg/mL	-4.1	0.7	3.93E-05
EFW 50μg/mL	-1.4	0.2	7.35E-06
PR 5μg/mL	-87.3	45.0	2.85E-02
PR 50μg/mL	-5.7	2.0	4.15E-03
40% 5μg/mL	-74.3	24.9	9.60E-03
40% 50μg/mL	-3.3	0.3	1.30E-07
100% 5μg/mL	-6.4	1.2	9.21E-05
100% 50μg/mL	-3.6	1.2	2.89E-03
C3G 2.4μg/mL	-20.5	3.6	2.05E-02
C3G 23.7μg/mL	-43.5	14.1	7.35E-03

TNFα	Average	S.E.M.	p-Value
EFW 5μg/mL	-1.2	1.1	4.37E-02
EFW 50μg/mL	-1.0	3.8	5.73E-01
PR 5μg/mL	-4.9	0.5	2.42E-05
PR 50μg/mL	-7.5	3.5	5.37E-02
40% 5μg/mL	-280.5	126.3	6.74E-02
40% 50μg/mL	-867.3	55.9	7.69E-06
100% 5μg/mL	-156.6	17.9	1.19E-04
100% 50μg/mL	2.3	1.6	2.08E-01
C3G 2.4μg/mL	-260.5	65.1	6.60E-03
C3G 23.7μg/mL	-2069.3	906.9	1.68E-02

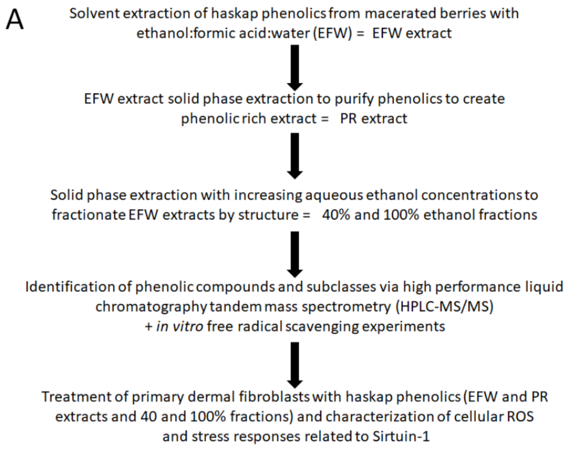

B

Classes of Compounds		EFW Extract	PR Extract	40% Fraction	100% Fraction
Phenolic Subclasses	Anthocyanins	X	X	X	
	Hydroxycinnamic Acids	X	X	X	
	Flavanols	X	X		X
	Flavonols	X	X		X
	Carbohydrates	X			
	Organic Acids	X			

Supplementary material Figure S1. Separation strategy to Generate Haskap Phenolic Fractions and their Composition. A) Strategy/workflow used to purify specific fractions (EFW, PR, 40% and 100%) from haskap berry variety Tundra and the methods used to identify specific phenolic subclasses contained within each. B) Table of identified phenolic subclasses identified in each of the fractions generated by the workflow.

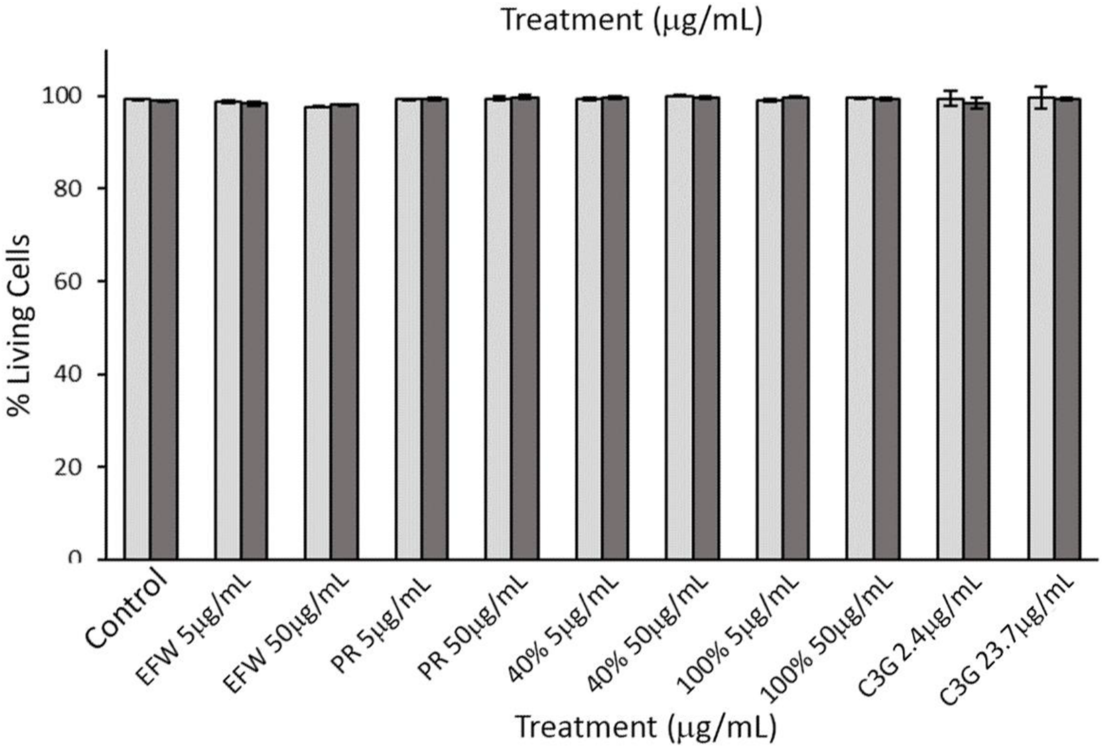

Supplementary material Figure S2. Phenolic Fractions and Extracts do not Result in Numbers of Trypan Blue Cells. The number of trypan blue negative 2DD (light grey bars) and NB1 hTERT (dark grey bars) fibroblasts was determined after 72 h treatment with Tundra variety haskap phenolic extracts (5 g/mL or 50 g/mL) and C3G (2.4 g/mL or 23.7 g/mL). Treatment abbreviations: EFW, ethanol:formic acid:water extract; PR, phenolic rich extract; 40%, 40% ethanol fraction; 100%, 100% ethanol fraction; C3G, cyanidin-3-*O*-glucoside. p-values = *p<0.10, **p<0.05, ***p<0.01.

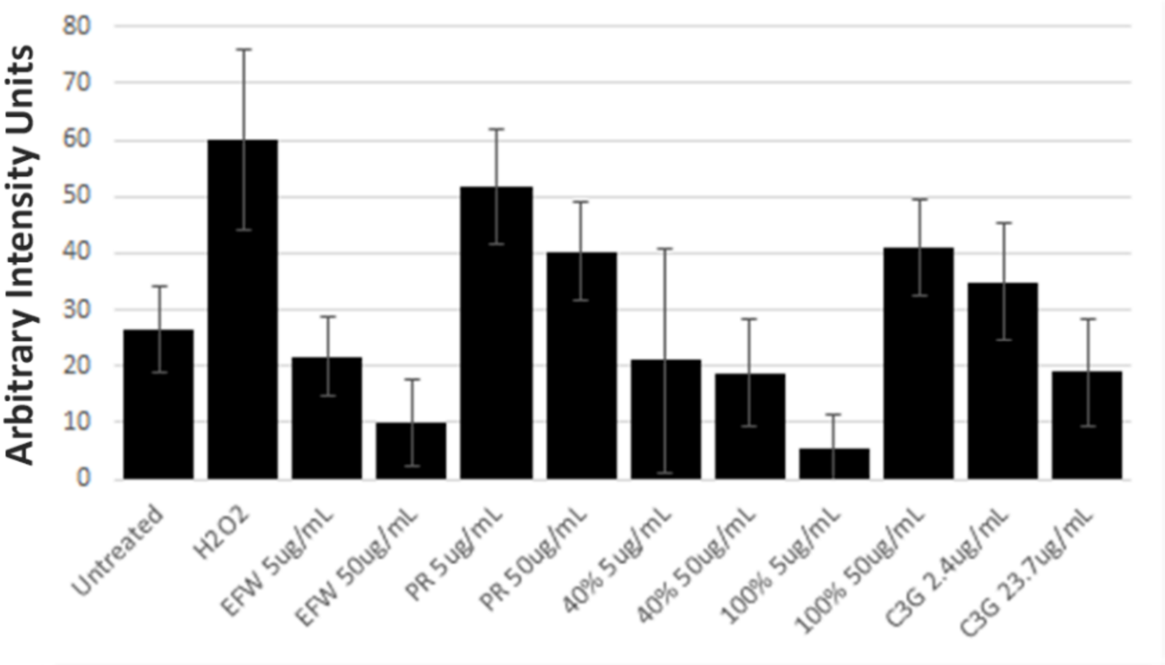

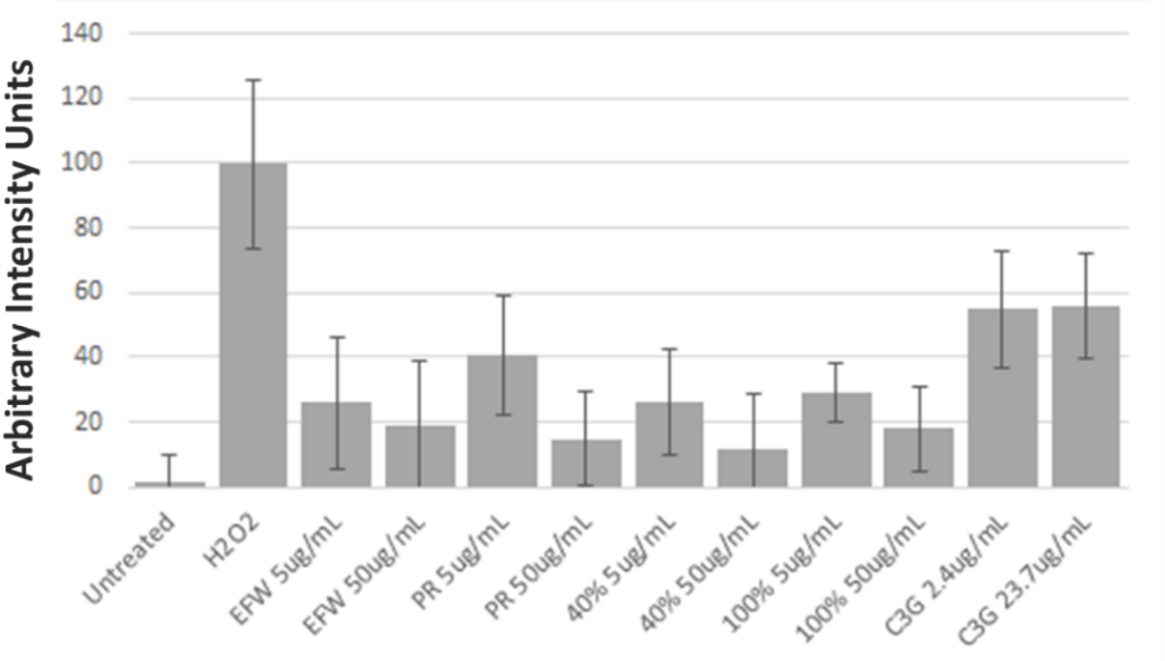

Supplementary material Figure S3. Phenolic Extracts and Fractions Decrease Intracellular Free Radicals. 2DD (A) and NB1hT (B) cells were treatment with Tundra variety haskap phenolic ex-tracts (5 g/mL or 50 g/ mL) and C3G (2.4 g/mL or 23.7 g/mL) for 72h (x-axis) followed by 30min exposure to 300 M H_2O_2. Following fixation cells were stained with Mitotracker Orange$_{TM}$. Images were collected with equivalent imaging conditions Signal quantification was performed (Y- Axis, arbitrary intensity units) by measuring to fluorescence in equal areas of the cytoplasm (including mitochondria). Intensity measures were averaged. Error bars = S.E.M.

	A Normalized to Control	B Normalized to Control	Average	S.E.M.
Control	1.0	1.0	1.0	0.0
EFW/5.0	1.2	1.5	1.4	0.1
EFW/50.0	1.1	1.0	1.1	0.1
PR/5.0	1.5	1.1	1.3	0.2
PR/50.0	1.6	0.8	1.2	0.4
40%/5.0	0.7	0.8	0.8	0.1
40%/50.0	0.4	0.8	0.6	0.2
100%/5.0	0.2	0.5	0.3	0.1
100%/50.0	0.3	0.4	0.3	0.1
C3G/2.4	1.6	3.2	2.4	0.8
C3G/23.7	1.0	1.8	1.4	0.4

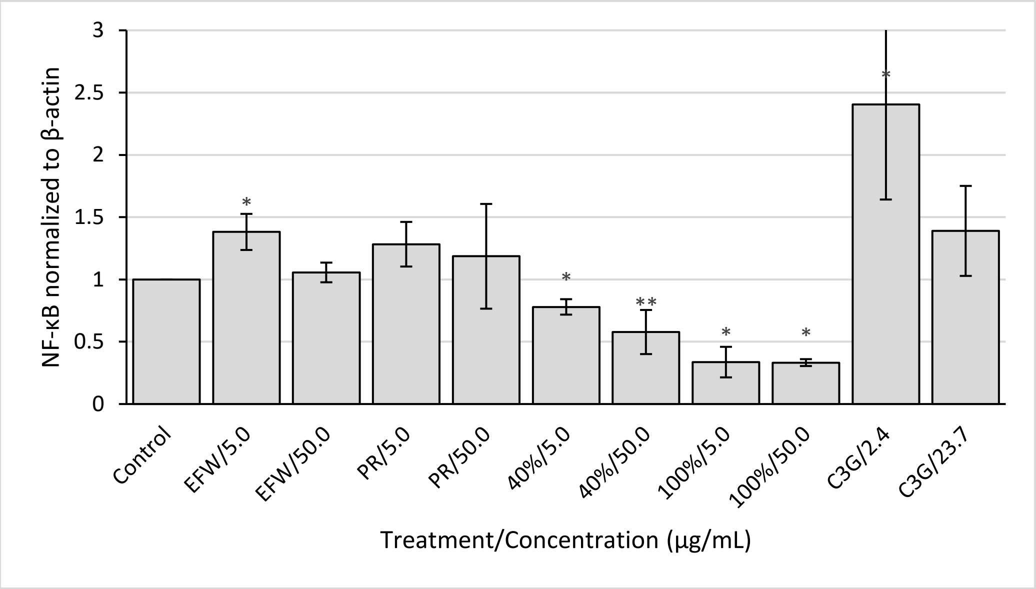

Supplementary material Figure S4. 2DD Cell p-p65 Normalization to Control Quantification Following Exposure to Haskap Phenolic Fractions. Western blot analysis for p-p65 and subsequent quantification of protein lysates from two biological replicates of 2DD cells treated with phenolic extraction and fractions. Left panel is the quantification values and the right panel demonstrates the fold changes observed. p-values = *p<0.10, **p<0.05, ***p<0.01.

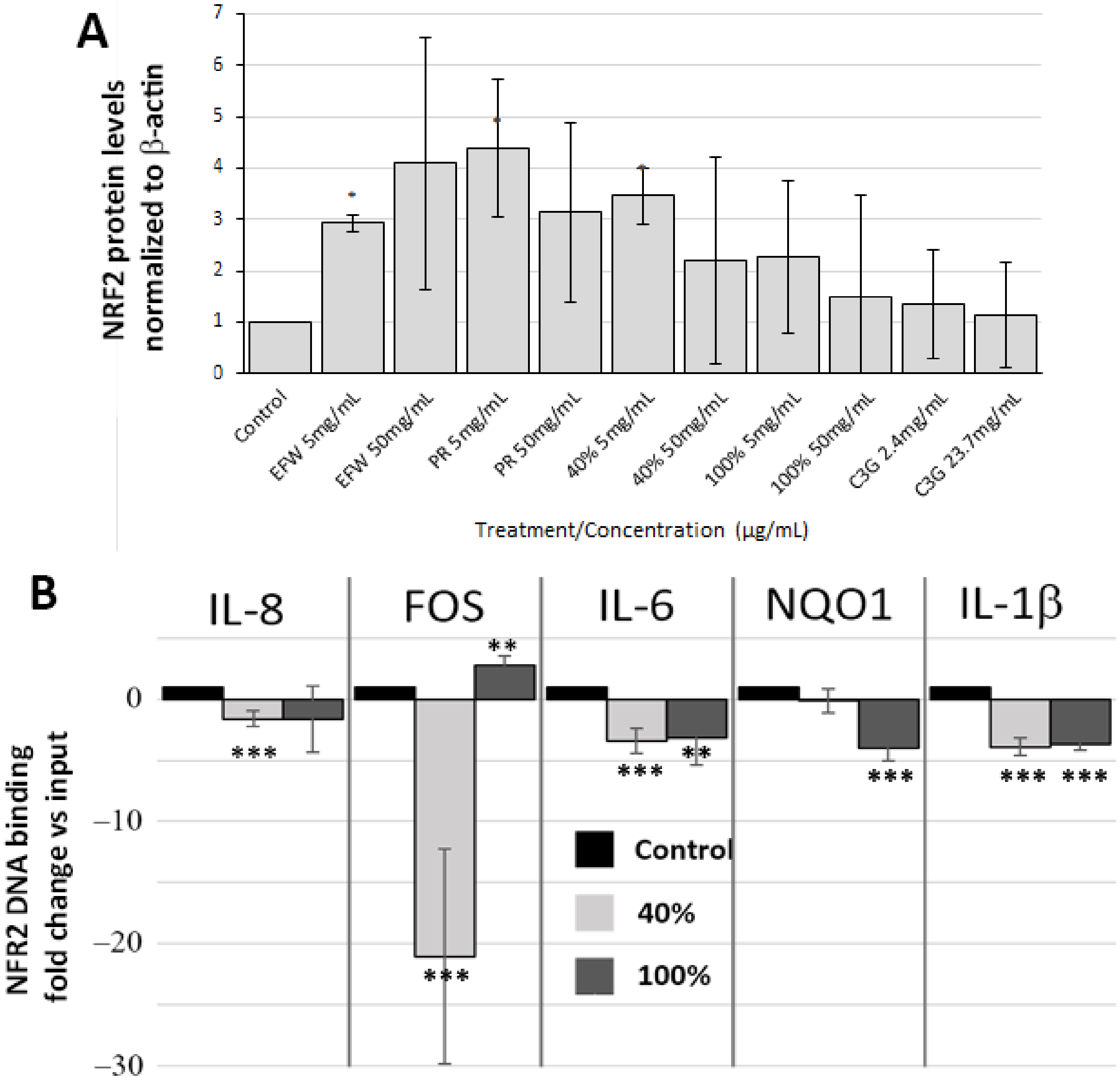

Supplementary material Figure S5. NRF2 Protein Levels Demonstrate Responsiveness to Haskap Phenolics but did not Increase DNA binding. NRF2 levels are increased in response to phenolic fractions but do not increase in DNA binding. Quantification of NRF2 protein levels in 2DD cells using β-actin as a normalization control. (B) ChIP and subsequent qPCR measured NRF2 binding within the IL8, FOS, IL6, NQO1 and IL-1β promoters. p-values = *$p<0.10$, **$p<0.05$, ***$p<0.01$.

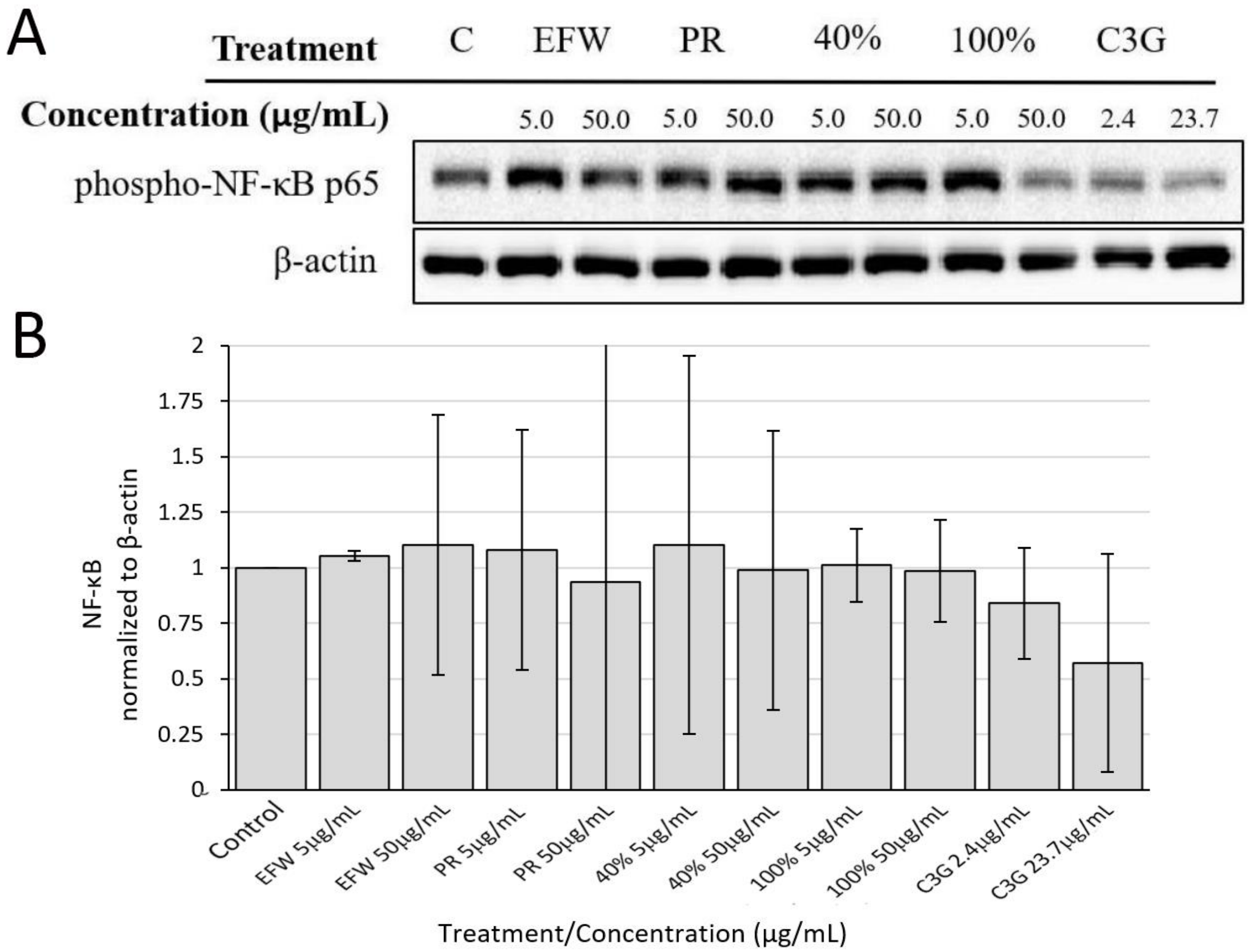

Supplementary material Figure S6. NF- B/p-p65 is No Longer Responsive to Phenolics Following SIRT1 Knockdown in Immortalized Fibroblasts. NB1hT cells were infected with lentivirus encoding siRNAs against SIRT1 and subsequently exposed to phenolic fractions (5 µg/ml or 50 µg/ml concentrations). Western blot analyses were performed on total cell protein extract p- p65. Equal protein equivalents were loaded and -actin was used as a normalization control for quan-tification. B) Quantification was performed by densitometry over two biological replicates. Error bars = S.E.M.
